# Trajectories of Change in an Open-access Internet-Based Cognitive Behavior Program for Childhood and Adolescent Anxiety: Open Trial

**DOI:** 10.2196/27981

**Published:** 2021-06-18

**Authors:** Sonja March, Philip J Batterham, Arlen Rowe, Caroline Donovan, Alison L Calear, Susan H Spence

**Affiliations:** 1 Centre for Health Research and School of Psychology and Counselling University of Southern Queensland Springfield Australia; 2 Centre for Mental Health Research Research School of Population Health The Australian National University Canberra Australia; 3 Centre for Health Research University of Southern Queensland Springfield Australia; 4 School of Applied Psychology Griffith University Mt Gravatt Australia; 5 Australian Institute for Suicide Prevention Griffith University Mt Gravatt Australia

**Keywords:** iCBT, child, adolescent, anxiety, online, trajectories of change

## Abstract

**Background:**

Although evidence bolstering the efficacy of internet-based cognitive behavioral therapy (iCBT) for treating childhood anxiety has been growing continuously, there is scant empirical research investigating the timing of benefits made in iCBT programs (eg, early or delayed).

**Objective:**

This study aims to examine the patterns of symptom trajectories (changes in anxiety) across an iCBT program for anxiety (BRAVE Self-Help).

**Methods:**

This study’s participants included 10,366 Australian youth aged 7 to 17 years (4140 children aged 7-12 years; 6226 adolescents aged 12-17 years) with elevated anxiety who registered for the BRAVE Self-Help program. Participants self-reported their anxiety symptoms at baseline or session 1 and then at the commencement of each subsequent session.

**Results:**

The results show that young people completing the BRAVE Self-Help program tend to fall into two trajectory classes that can be reliably identified in terms of high versus moderate baseline levels of anxiety and subsequent reduction in symptoms. Both high and moderate anxiety severity trajectory classes showed significant reductions in anxiety, with the greatest level of change being achieved within the first six sessions for both classes. However, those in the moderate anxiety severity class tended to show reductions in anxiety symptoms to levels below the elevated range, whereas those in the high symptom group tended to remain in the elevated range despite improvements.

**Conclusions:**

These findings suggest that those in the high severity group who do not respond well to iCBT on a self-help basis may benefit from the additional support provided alongside the program or a stepped-care approach where progress is monitored and support can be provided as necessary.

## Introduction

Anxiety is an increasingly common childhood mental health condition, with potentially significant life-long repercussions for those who do not access early treatment [1]. Internet-based or eHealth interventions are recommended for encouraging the primary prevention and early intervention for mental illness [2]. In particular, the evidence base for internet-based cognitive behavior therapy (iCBT) in the treatment of childhood anxiety has been growing continuously, with the best outcomes observed when delivered with therapist support [3-8]. More recently, research has demonstrated the feasibility of self-help iCBT for childhood anxiety as a means of achieving widespread service implementation in real-world clinical and community contexts [9]. Self-help iCBT, which does not rely on any therapist support, has the potential to offer evidence-based mental health assistance in a timely and effective manner and in a way that overcomes barriers to accessibility, cost, stigma, anonymity, and shortages of health care professionals [10]. Several successful iCBT platforms have been developed and validated internationally for children and young people (eg, BIP Anxiety [11,12] and SPARX [13]) and adults (eg, MindSpot Clinic [14], This Way Up [15], and Shuti [16,17]).

The BRAVE Self-Help program is the only widely disseminated iCBT program for childhood anxiety, which is made publicly available, free of charge, to all children and adolescents in Australia. Although the preliminary effects of this self-help iCBT program appear somewhat weaker than those found for the therapist-supported iCBT version [9], clinically meaningful improvements are still made by a large proportion of anxious youth who engage in and complete the program, further demonstrating its potential as a population-level, early intervention. Specifically, it was demonstrated that for those users who completed six or more self-help iCBT sessions, there was a moderate to large reduction in anxiety (Cohen *d*=0.81 and Cohen *d*=0.87 for adolescents and children, respectively [9]). Furthermore, for those who had completed nine sessions, approximately 57.7% (94/163) achieved recovery into nonelevated levels of anxiety and 54.6% (89/163) showed statistically reliable reductions in anxiety [9]. However, anxiety was not measured at every session, and the amount of change made from session to session was not explicitly tested. Therefore, it is unclear when reductions in anxiety actually occurred and for whom.

Very little research has been conducted on the timing of benefits made in iCBT programs (eg, early or delayed). March et al [9] demonstrated that users’ anxiety was reduced after only three self-help iCBT sessions (Cohen *d*=0.59), suggesting that change may occur early and rapidly. Furthermore, in their examination of brief and full versions of a cognitive behavioral therapy (CBT) website (MoodGym) for adults with elevated depression, Christensen et al [18] found that a single module of CBT was insufficient to reduce depression symptoms, but extended CBT (approximately three sessions) was associated with greater improvements. Importantly, however, they also found that programs longer than three sessions were not necessarily associated with greater improvement [18]. There is also some evidence from face-to-face interventions that can provide potential insight into the ideal dosage and response trajectories. In one study of face-to-face CBT for anxious youth, a nonlinear symptom trajectory was reported, whereby a rapid response was evident over the first six sessions, with tapering anxiety reductions over the remainder of the intervention [19]. In another study examining the separate trajectories of anxiety and depressive symptoms over the course of a transdiagnostic treatment for adolescents, Queen et al [20] found that, on average, participants’ total anxiety scores reduced steadily during treatment (by 4.76 units every 8 weeks) and then slowed during the follow-up period (reduced by only 1.48 units every 8 weeks). Together, the evidence to date suggests that a reduction in symptoms appears to commence after at least three sessions of iCBT [9,18]. However, a session-by-session trajectory across iCBT programs for children and adolescents has yet to be examined.

We examined patterns of symptom trajectories (changes in anxiety) across BRAVE Self-Help program sessions to determine whether BRAVE Self-Help produces a gradual and linear impact on anxiety symptoms or whether the greatest impacts are made early in treatment and to determine distinct trajectories of anxiety symptoms within BRAVE Self-Help participants. Such information will help in determining ideal doses of self-help iCBT and whether small doses of treatment are sufficient. Given that variability is likely to be present in how young people respond to self-help interventions, identifying factors associated with different trajectories may assist in understanding the mechanisms through which the program works and may inform targeting and tailoring of the program. Thus, we aim to identify subgroups of participants (including identifying common demographic and clinical characteristics of these subgroups) based on latent change trajectories in anxiety scores in response to BRAVE Self-Help. Previous studies have used this analytic approach to determine the trajectories of depressive symptoms and suicidal ideation in internet-based interventions [17,21], and in longitudinal studies examining trajectories of anxiety symptoms [22,23]; however, this approach is yet to be applied for examining responses to anxiety treatment in the context of a web-based program for young people. The identification of key demographic and clinical variables that predict subgroup membership has the potential to inform the effective delivery of supported interventions, tailored, or alternative treatments. In this way, the results will inform recommendations regarding the use of iCBT within the context of population-level models of care that are open to everyone and are typically not monitored by health care professionals.

## Methods

### Intervention

BRAVE Self-Help is an interactive internet-based iCBT program for preventing and treating anxiety among youth. The program, described extensively elsewhere [9,24], is offered as an open-access web-based program targeting young Australians aged 7 to 17 years. It comprises 10 sessions of 30 to 60 minutes each, with two additional booster sessions that can be completed as revision modules. Sessions include CBT techniques incorporated into interactive web-based activities focused on psychoeducation, recognition of physiological symptoms of anxiety, relaxation training, cognitive strategies of coping statements and cognitive restructuring, graded exposure, problem-solving approaches, self-reinforcement, and relapse prevention. Each session comprised pages that included the presentation of information or material, examples of technique application, activities to facilitate the knowledge acquisition and application of skills to the young person’s circumstances, quizzes to consolidate learning, and homework activities to promote the application of skills in real-world contexts. The self-help program has previously demonstrated its acceptability and feasibility [9].

Depending on age, participants completed either the child program (7-12 years) or the adolescent program (12-17 years), with 12-year-olds given a choice of either program. Although there were accompanying parent programs, these were not included in this study. BRAVE Self-Help is delivered without any therapist support, and there are no timing restrictions between sessions; however, the sessions must be completed in a predetermined sequence. Automatic reminders to complete sessions are sent to a young person via email.

### Participants

Participants included 10,366 Australian youth aged 7 to 17 years with elevated anxiety (4140 children aged 7-12 years; 6226 adolescents aged 12-17 years) who registered for the BRAVE Self-Help program between July 1, 2014, and October 26, 2018. Table 1 presents a full summary of the baseline characteristics of participants according to program grouping.

**Table 1:** BRAVE Self-Help baseline participant characteristics (N=10,366).

Characteristics			Child program (n=4140)	Adolescent program (n*=*6226)	Total participants (N=10,366)
Age (years), mean (SD)			9.34 (1.48)	14.55 (1.66)	12.47 (3.01)
**Gender, n (%)**					
	Male		2192 (52.95)	1480 (23.77)	3672 (35.42)
	Female		1948 (47.05)	4532 (72.79)	6480 (62.52)
	**Other**		0 (0)	214 (3.44)	214 (2.06)
		Transgender or transsexual	0 (0)	40 (0.64)	40 (0.39)
		Transgender or transsexual	0 (0)	12 (0.19)	12 (0.12)
		Genderqueer	0 (0)	59 (0.95)	59 (0.57)
		Androgynous	0 (0)	28 (0.45)	28 (0.27)
		None of the above	0 (0)	75 (1.21)	75 (0.71)
**Remoteness area, n (%)**					
	Major cities		2324 (56.14)	3559 (57.16)	5883 (56.75)
	Inner regional		1013 (24.47)	1424 (22.87)	2437 (23.51)
	Outer regional		499 (12.05)	712 (11.44)	1211 (11.68)
	Remote		111 (2.68)	141 (2.27)	252 (2.43)
	Very remote		38 (0.92)	20 (0.32)	58 (0.56)
	Missing		155 (3.74)	370 (5.94)	525 (5.07)
Number of sessions completed, mean (SD)			3.04 (2.98)	2.04 (2.51)	2.39 (2.67)
**Baseline anxiety**					
	CAS-8^a^, mean (SD)		14.16 (3.04)	15.75 (3.42)	15.11 (3.36)
	Elevated, n (%)		2275 (54.95)	2819 (45.28)	5094 (49.14)
	Clinical, n (%)		1865 (45.05)	3407 (54.72)	5272 (50.86)

^a^CAS-8: Children’s Anxiety Scale 8-item.

The BRAVE Self-Help program is an open-access intervention offered to young people and families throughout Australia; thus, registration does not require referral from a health care professional. Children and adolescents who registered for the program were referred by school-based professionals (3480/10,366, 33.57%); referred by external health professionals (1973/10,366, 19.03%); referred by parents, friends, or family members (1330/10,366, 12.83%); referred through *beyondblue* (645/10,366, 6.22%); self-referred through internet searching (840/10,366, 8.1%); or referred through other means (eg, word-of-mouth, radio, magazine, or advertising; 676/10,366, 6.52%). A full breakdown of referral sources is provided in Multimedia Appendix 1.

Eligible participants were required to have registered for the BRAVE Self-Help program between July 2014 and October 2018 and to have completed the Children’s Anxiety Scale 8-item (CAS-8) [25] during the registration process. All participants had access to BRAVE Self-Help (single-cohort longitudinal design); there was no randomization or comparison condition. Participants were not required to demonstrate symptomatic levels of anxiety to register for the program; however, only those with elevated anxiety were included in this study. Of the participants included in this study, 49.14% (5094/10,366) showed elevated levels of anxiety (CAS-8 ≥84th percentile or T-score ≥60) and 50.89% (5272/10,366) showed clinical levels of anxiety (CAS-8 ≥96th percentile or T-score ≥65). As the primary objective was to examine the trajectories of session completion and reductions in anxiety, there were no inclusion criteria regarding the minimum number of completed program sessions.

### Measures

All measures were embedded within the BRAVE Self-Help program. Further data were collected via BRAVE program analytics regarding adherence to the program.

#### Sociodemographics

Basic demographic information, including age, gender, and location (postcode), was collected during the registration process. Age was measured in years, and the residential location was assessed using postcode. Postcode data were coded according to the Australian Standard Geographic Classification system [26], and participants were grouped into the categories of major cities, inner regional, outer regional, remote, and very remote locations. In terms of gender, participants were able to select from male, female, transgender or transsexual, transgender or transsexual, genderqueer, androgynous, or other. For the purposes of analysis, gender was subsequently recoded into male, female, and other.

#### Anxiety Severity

Anxiety levels were measured using the CAS-8 [25]. The CAS-8 comprises eight items asking the young person to rate how often each item (eg, “I worry what other people think of me”) applied to them. Items are rated on a 4-point scale ranging from 0 (*never*) to 3 (*always*). Items are summed to produce a total score ranging from 0 to 24, with higher scores indicating greater levels of anxiety.

The CAS-8 has been used in large-scale school-based prevention program trials with subsequent population-level, gender-standardized norms for comparison [25]. Scores ≤83rd percentile (≤T-score 59: CAS-8 score ≤9 for males and 11 for females) are considered *normal*; scores ≥84th percentile (above T-score 60: CAS-8 score ≥10 for males and 12 for females) are considered indicative of *elevated anxiety* and scores ≥94th percentile (above T-score 65: CAS-8 score ≥13 for males and 16 for females) indicate clinical levels of anxiety. For this study, and as per procedures of March et al [9], for other gender, scores ≥11 were considered indicative of *elevated* and scores ≥15 were considered indicative of *clinical* anxiety. The CAS-8 was completed by participants at baseline or session 1 and then at the commencement of each subsequent session (ie, up to nine time points after baseline). For example, if a participant had completed five sessions, they would have CAS-8 data at baseline and sessions 2, 3, 4, and 5. The CAS-8 has demonstrated good psychometric properties, with a reliability coefficient α of .89 in previous studies [25] and an average internal consistency across data collection points of 0.83 in this study.

#### Program Adherence

Program adherence was measured as the number of sessions completed by each participant over a period of 20 weeks from the date of registration. The number of sessions completed was automatically recorded by the program.

### Procedure

Data for this study were collected as a part of a large community effectiveness trial of the BRAVE Self-Help program (ethical approval H13REA264 from the governing university). During the registration process, participants were required to read a developmentally appropriate web-based participant information sheet and provide informed consent. Children aged below 16 years were also required to obtain parental consent to continue with registration. After providing consent, participants created a profile and completed an initial questionnaire, including basic demographic data (age, gender, location, referral source, and email contact address) and baseline anxiety assessment (CAS-8). Participation in the program was voluntary, and participants could choose to provide anonymous information (eg, nickname) if they chose. They could also discontinue using the program or withdraw from the study at any time without adverse consequences.

**Figure 1:**
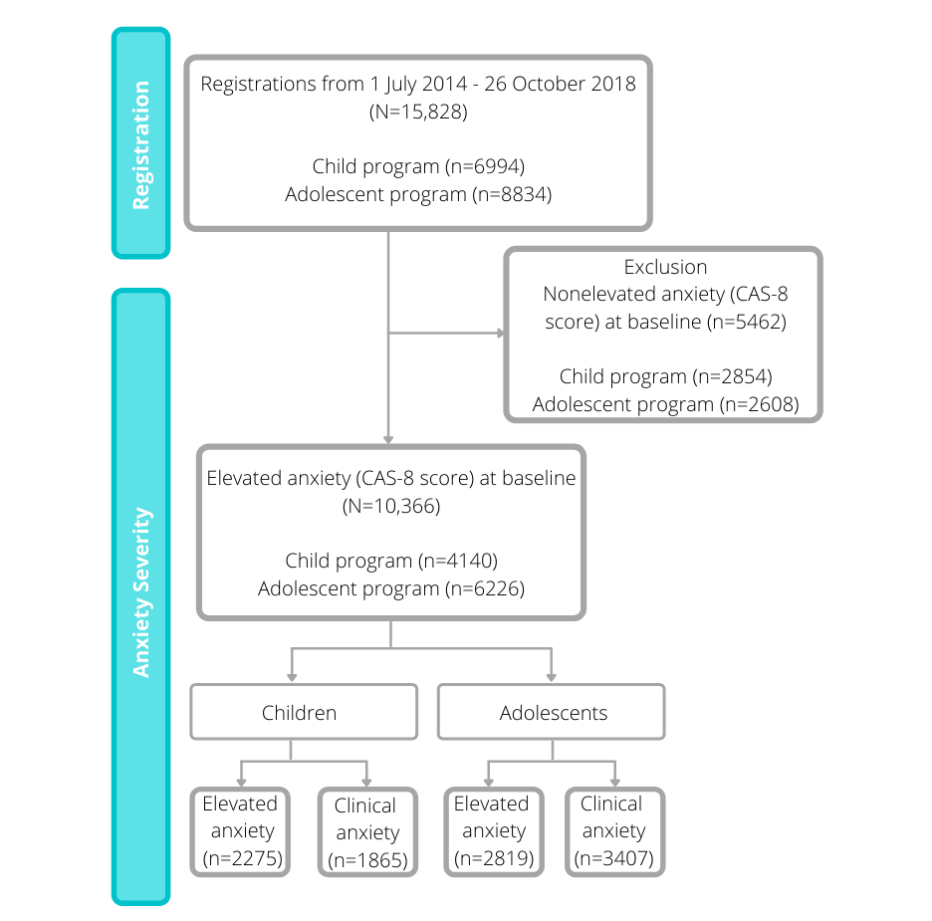
Participant inclusion in the BRAVE Self-Help program. CAS-8: Children’s Anxiety Scale, 8-item.

Participant data were recorded for a 20-week period from the date of registration, thus allowing sufficient time for the completion of the 10 sessions. Figure 1 provides a visual representation of the selection process for the inclusion and exclusion of data and the final sample included in the study.

### Analytic Strategy

Trajectories of anxiety symptoms based on CAS-8 scores were identified using growth mixture models (GMMs), with intercept, linear change, and quadratic change estimated using all available data from the baseline assessment and up to nine subsequent measurement occasions. GMM was used to identify latent classes of participants with distinct longitudinal trajectories, with all available data included in the models [27]. The GMM approach is a form of latent class analysis that identifies distinct subgroups, combined with growth modeling that estimates linear and quadratic trajectories over time. The methodology classifies the heterogeneity of individual trajectories in symptoms over time into a discrete number of latent classes. Models with between one and five latent classes were tested to identify an optimal number of latent classes based on a significant Bootstrapped Likelihood Ratio Test (BLRT) [28]. We also considered the size of each latent class, as models with classes that represent <5% of the total sample are difficult to identify [28]. After selecting an optimal number of classes, predictors of latent class membership were tested using logistic regression models to identify participants’ characteristics (eg, baseline anxiety severity, gender, age, and geographic location), who showed differential responses to the intervention, and to characterize the relationship between adherence and response to the BRAVE Self-Help program. GMM analyses were conducted using Mplus version 7 (Muthén & Muthén), whereas descriptive and regression analyses were conducted using SPSS version 25 (IBM Corp).

## Results

### Program Adherence

**Figure 2:**
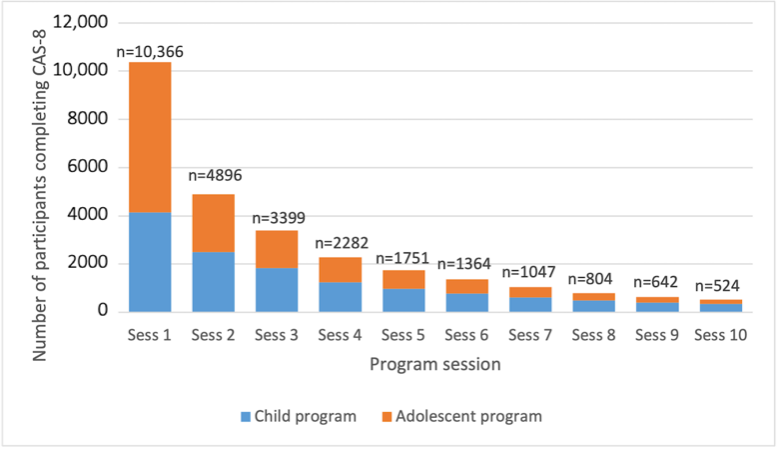
Rates of program adherence. CAS-8: Children’s Anxiety Scale, 8-item.

The number of participants completing each session decreased throughout the program. As indicated in Figure 2, only half of the participants completed two sessions of the program.

### Trajectories of Anxiety Symptoms

**Figure 3:**
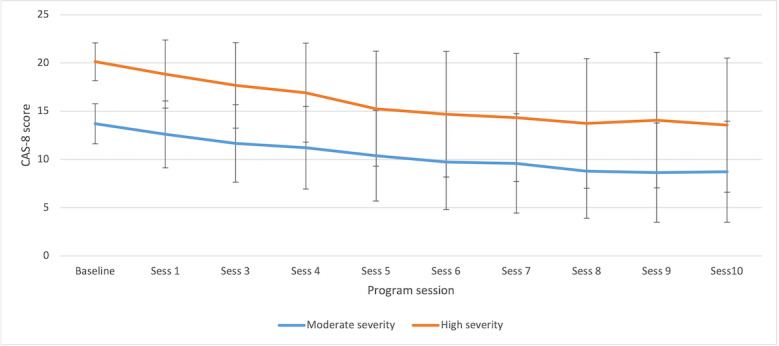
Trajectories of anxiety symptoms for the two latent classes of high and moderate anxiety severity based on observed mean (SD). CAS-8: Children’s Anxiety Scale, 8-item.

The GMM with two classes had a significantly better fit than a single-class solution based on the BLRT statistic (*P*<.001); however, the BLRT for a three-class model was not significant as compared with the two-class model (*P*=.99). Furthermore, in the three-class model, one of the classes accounted for 0.7% (73/10,366) of the sample. Consequently, a two-class model was selected. Scores on the CAS-8 for the two total classes are shown in Figure 3. On the basis of the observed trajectories, the two classes were labeled as *high anxiety severity* (*HAS*; 2280/10,366, 21.99% of the sample, shown in orange), and *moderate anxiety severity* (*MAS*; 8086/10,366, 78.01% of the sample, shown in blue). On the basis of the GMM model, the intercept for the CAS-8 scores in the HAS group was 19.72 (SE 0.09), with a negative slope (estimate=−1.39; SE 0.11; *P*<.001). The intercept for the MAS group was 13.75 (SE 0.04), with a negative slope (estimate=−0.97; SE 0.04; *P*<.001). A quadratic relationship was also evident in both the HAS (estimate=0.08; *P*<*.*001) and MAS (estimate=0.05; *P*<*.*001) groups, showing that the rate of change in CAS-8 scores decreased over time for both classes.

### Predictors of Class Membership

A logistic regression model was used to identify the factors associated with class membership. The outcomes are presented in Table 2. Teen program participants had 31% greater odds of being in the HAS class than those receiving the child program (*P*<*.*001). For every year increase in age, participants were 4% more likely to fall in the HAS class (*P=.*007). Females had twice greater odds of being in the HAS class than males (*P*<.001). Participants of other gender had 47% greater odds of being in the HAS class than females (*P=.*007). The effects of remoteness of residence were not significant (Table 2). In terms of program adherence, those participants who completed a greater number of sessions were less likely to be classified as HAS, with the likelihood of being in the HAS class decreasing by 3% with the completion of each additional session (*P*=.01). There were no effects on the date of registration or the number of activities completed in session 1.

**Table 2:** Logistic regression model of class membership: odds of having a high compared with moderate anxiety severity class trajectory.

Variable			Estimate (SE)		Chi-square (*df*)		*P* value		Odds ratio (95% CI)
Program: teen versus child			0.27 (0.05)		29.9 (1)		*<.001* ^a^		1.31 (1.19 to 1.44)
Age at registration			0.04 (0.02)		7.2 (1)		*.007*		1.04 (1.01 to 1.07)
**Gender**					148.5 (2)		*<.001*		
	Male versus female	−0.69 (0.06)		135.8 (1)		*<.001*		0.50 (0.45 to 0.56)	
	Other versus female	0.38 (0.14)		7.2 (1)		*.007*		1.47 (1.11 to 1.95)	
**State of residence**					17.3 (8)		*.03*		
	Missing versus SA^b^	−0.44 (0.50)		0.8 (1)		.38		0.64 (0.24 to 1.71)	
	ACT^c^ versus SA	−0.10 (0.19)		0.3 (1)		.57		0.90 (0.62 to 1.30)	
	NSW^d^ versus SA	−0.23 (0.09)		6.0 (1)		*.01*		0.79 (0.66 to 0.95)	
	Northern Territory versus SA	0.18 (0.28)		0.4 (1)		.52		1.19 (0.69 to 2.05)	
	Queensland versus SA	−0.28 (0.10)		8.5 (1)		*.004*		0.75 (0.62 to 0.91)	
	Tasmania versus SA	−0.40 (0.18)		5.0 (1)		.*02*		0.67 (0.47 to 0.95)	
	Victoria versus SA	−0.33 (0.10)		11.3 (1)		*.001*		0.72 (0.59 to 0.87)	
	Western Australia versus SA	−0.31 (0.11)		8.4 (1)		.*004*		0.73 (0.59 to 0.91)	
**Remoteness area**					4.0 (5)		.54		
	Missing versus major city	0.18 (0.117)		2.8 (1)		.10		1.20 (0.97 to 1.48)	
	Inner regional versus major city	−0.01 (0.064)		0.0 (1)		.93		0.99 (0.88 to 1.12)	
	Outer regional versus major city	−0.01 (0.084)		0.0 (1)		.90		0.99 (0.85 to 1.16)	
	Remote area versus major city	−0.05 (0.168)		0.1 (1)		.78		0.96 (0.69 to 1.32)	
	Very remote area versus major city	−0.37 (0.398)		0.9 (1)		.34		0.69 (0.32 to 1.49)	
Date of registration			−0.01 (0.021)		7.4 (1)		.45		0.99 (0.95 to 1.02)
Number of sessions completed			−0.03 (0.011)		12.2 (1)		*.01*		0.97 (0.95 to 0.99)
Activities completed in session 1			0.00 (0.005)		1.5 (1)		.40		1.00 (0.99 to 1.01)
Constant			4.49 (8.44)		0.3 (1)		.59		N/A^e^

^a^Italics indicate statistical significance (*P*<.05).

^b^SA: South Australia.

^c^ACT: Australian Capital Territory.

^d^NSW: New South Wales.

^e^N/A: not applicable.

## Discussion

### Principal Findings

This study examined the trajectories of responses to a web-based, open-access, self-help intervention for youth anxiety. The results suggested two classes of trajectories that could be distinguished by high versus moderate baseline levels of anxiety and levels of change over time. Irrespective of trajectory, participants, on average, obtained benefits from the program, with participants generally showing significant linear reductions in anxiety. From a baseline average of around 20 (>98th percentile for females and males), those in the HAS trajectory class showed reduced anxiety scores of 6.58 points (or 5.68 points based on model estimates that accounted for missing data) on the CAS-8; however, this level remained within the elevated range. In contrast, the MAS trajectory class showed reductions of 3.87 points (or 4.31 points based on model estimates) on the CAS-8 from a baseline of 12.6 (84th percentile for females; 94th percentile for males) to a level in the nonelevated range. Indeed, on average, those in the MAS class obtained a clinically meaningful benefit (scores within the nonelevated range) by session 6.

The finding that the HAS class tended to remain in the elevated range of anxiety scores despite showing significant reductions from a very high starting point suggests that at least a proportion of this group may require additional assistance over and above the self-help iCBT program. The finding also revealed that the HAS class completed fewer sessions than their MAS counterparts. It is possible that lower levels of perceived symptom improvement among participants in the HAS class contributed to lower motivation to continue engaging with the program, or a greater difficulty completing sessions due to ongoing symptom severity and difficulty implementing strategies. Thus, a therapist-supported model or stepped-care approach may be beneficial for this group, whereby HAS participants either receive additional support from the outset or are monitored (in terms of anxiety reduction and session compliance) and are referred for additional therapist support as necessary. HAS participants could be easily identified at the beginning of the program (due to higher initial scores), and subsequently referred, monitored, and provided with additional support or intervention elements if their response to the program was poor. Preliminary results from case studies of a stepped-care model of BRAVE highlight the potential of this approach [29].

The study results also suggested that the rate of anxiety reduction decreased over time for both trajectory classes, with greater reductions occurring in the first six sessions than in later sessions. Importantly, in line with the findings of Christensen et al [18], the results showed that young people can show significant reductions in symptoms after brief iCBT programs. The findings are also partially consistent with the findings of Chu et al [19] for face-to-face CBT for youth anxiety; however, Chu et al [19] showed a more rapid initial decrease and a clearer plateau than this study. The results are also consistent with those of Queen et al [20], who demonstrated a large reduction in symptoms during the first eight weeks of face-to-face treatment and a far smaller drop in symptoms in the second eight weeks of follow-up treatment. It would seem that around six sessions of iCBT may be sufficient for many users, with the option of further session completion for those who choose to do so, or for whom poor treatment response suggests they may be indicated. Importantly, with the knowledge that six sessions may be sufficient for many users, this provides an opportunity to develop shorter intervention programs that may be more appealing and motivating for young people and ultimately attract more users.

In terms of predictors of class membership, the study found that adolescents and females were more likely to be in the HAS class compared with MAS class than children and males. These findings are perhaps unsurprising given that anxiety levels are known to increase with age and are higher among women [30]. Given that the HAS class tended to complete fewer sessions and were less likely to reach a nonelevated status, it is important that the greater representation by females and adolescents is considered to ensure that the needs of these participants are being considered in program design and delivery. For example, our previous research indicated that adolescents are more likely to complete the program alone without parental support. It is possible that additional forms of support, whether from a web-based therapist, school teacher, peer coach, or even automated chatbot could enhance treatment outcomes for adolescents. It may also be feasible to build web-based reward systems designed for adolescents that provide enhanced support to increase motivation and program adherence. Earlier dropouts among HAS participants may suggest that those with elevated symptoms found it more difficult to engage in the program. Nevertheless, the two classes had similar levels of initial engagement (based on activity completion), suggesting that other factors, such as perceived treatment efficacy, may have influenced adherence.

The implications of this research’s findings are not limited to the BRAVE program and provide useful insights for other web-based interventions. For example, when there is a broad range of users who engage with an internet intervention, with diverse profiles, particularly related to symptom severity, the results of this study indicate that care should be taken to consider the needs of these subgroups. Particular consideration should be given to whether the service model is appropriate for them, whether the service needs to be tailored to profiles (eg, with additional support), or whether the service needs to be targeted to those who will benefit the most. However, it is important to note that there are drawbacks to targeting more narrowly—there is still a clear demand for the BRAVE program even among those in the high severity class, which suggests that many young people with anxiety may not be receiving the care they need from other sources (whether web-based or in-person).

### Strengths and Limitations

There are a number of clear strengths of this study. It had a large sample size, included both children and adolescents, used naturalistic data from a clinical service without stringent eligibility criteria, and collected symptom-level data throughout the completion of the program. However, alongside these strengths, this study has a number of limitations. High attrition was observed, and although models were robust to missing data, dropout may be associated with treatment response. All outcome measures were self-reported; thus, there was no clinical verification of the symptom level. Furthermore, as the data were collected in the context of clinical service delivery, there was no control group. This makes it unclear whether factors external to the program may have influenced the trajectories and whether the reductions in anxiety were attributed to the treatment or were the result of spontaneous recovery over time. Finally, limited psychosocial factors were measured; thus, other factors, such as ruminative style, personality, social support, and previous exposure to treatment, may have influenced the observed trajectories.

### Future Research Directions

Recent worldwide events have resulted in a significant shift in mental health care service needs, as numerous lockdowns during the COVID-19 pandemic severely impacted face-to-face mental health service access and provision. This subsequently led to the conversion of many services to telehealth, videoconferencing, and other digital modalities to provide continuity of care. With an increased access to digital mental health services, and likely increased experiences of distress at the community level, it will be important to examine whether the use of the BRAVE Program changes throughout and after COVID-19. Future research should examine whether users presenting to the web-based program during and after the pandemic present with different profiles and symptom presentations and whether their experience with the web-based program leads to similar outcomes as standard BRAVE program users.

### Conclusions

Young people completing the BRAVE Self-Help program tend to fall into two trajectory classes that can be reliably identified in terms of high versus moderate baseline levels of anxiety and subsequent reductions in symptoms. Both classes showed significant reductions in anxiety, with the greatest level of change being achieved within the first six sessions for both classes. Those in the MAS class tended to show reductions in anxiety symptoms to levels below the elevated range, whereas those in the high symptom group tended to remain in the elevated range despite improvements. Thus, those in the high severity group who do not respond well to iCBT on a self-help basis may benefit from a stepped-care approach or additional support.

## Appendix

Multimedia Appendix 1 Breakdown of referral sources to the BRAVE self-help program (N=10,366).

## Abbreviations

BLRT: Bootstrapped Likelihood Ratio Test
CAS-8: Children’s Anxiety Scale 8-item
CBT: cognitive behavioral therapy
GMM: growth mixture model
HAS: high anxiety severity
iCBT: internet-based cognitive behavior therapy
MAS: moderate anxiety severity
