# Supplementary material for: Trajectories of Change in an Open-access Internet-Based Cognitive Behavior Program for Childhood and Adolescent Anxiety: Open Trial
Source: JMIR Ment Health. 2021 Jun 18;8(6):e27981. doi: 10.2196/27981 (PMC8277375; doi:10.2196/27981)
Supplement: Multimedia Appendix 1 [file mental_v8i6e27981_app1.docx]

## Multimedia Appendix 1

Breakdown of BRAVE Self-Help referral sources (n=10,366).

| **Referral Source n (%)** | | **Child Program**  ***n* = 4,140** | **Adolescent Program**  ***n =* 6,226** | **Total Participants**  ***n* = 10,366** |
| --- | --- | --- | --- | --- |
| School-based Professionals | | 1307 (31.57) | 2173 (34.90) | 3480 (33.57) |
| Parent/Family member | | 599 (14.47) | 731 (11.74) | 1330 (12.83) |
| Beyond Blue | | 165 (3.99) | 480 (7.71) | 645 (6.22) |
| Self-referral | | 430 (10.39) | 410 (6.59) | 840 (8.10) |
| External Health Professionals | | 867 (20.94) | 1106 (17.76) | 1973 (19.03) |
|  | GP/Family doctor | 134 (3.24) | 211 (3.39) | 345 (3.33) |
|  | Psychologist | 537 (12.97) | 568 (9.12) | 1105 (10.66) |
|  | Social Worker | 51 (1.23) | 102 (1.64) | 153 (1.48) |
|  | Occupational Therapist | 38 (0.92) | 36 (0.58) | 74 (0.71) |
|  | Nurse | 12 (0.29) | 36 (0.58) | 48 (0.46) |
|  | Other | 78 (1.88) | 108 (1.74) | 186 (1.79) |
|  | Don’t Know | 17 (0.41) | 46 (0.74) | 63 (0.61) |
| Other referral (e.g. word-of-mouth, radio, magazine, advertising) | | 352 (8.50) | 324 (5.20) | 676 (6.52) |
| Unknown | | 420 (10.15) | 1002 (16.09) | 1422 (13.72) |
